# The retail brand personality—Behavioral outcomes framework: Applications to identity and social identity theories

**DOI:** 10.3389/fpsyg.2022.903170

**Published:** 2022-08-16

**Authors:** Ya-Hui Kuo

**Affiliations:** ^1^Institute of International Business, National Cheng Kung University, Tainan, Taiwan; ^2^School of Retailing and Consumer Sciences, University of Arizona, Tucson, AZ, United States

**Keywords:** retail brand personality, self-congruity, brand identification, WOM, patronage intention, shopping conspicuousness situation

## Abstract

This study applies identity and social identity theories to develop and test a framework in which retail brand personality influences consumer outcomes [i.e., positive word-of-mouth (WOM) about and patronage intention toward the retailer] through public and/or private self-congruity, strengthened by shopping conspicuousness situation, and retail brand identification (RBI). This is the first study to include social shopping situations to study brand personality and self-congruity. A questionnaire with a 2 (retailer image format) × 2 (shopping situation conspicuousness) between-subjects design was conducted on a sample of US consumers. Structural equation modeling was used to test the hypotheses. The findings suggest a framework in which *Genuine*, the most influential dimension of retail brand personality, predicted outcome behaviors both directly and indirectly through self-congruities and RBI. The high shopping conspicuousness situation strengthened the relationship between public self-congruity and the overall RBI. The concept of RBI provides an additional theoretical perspective for guiding future research on shopper–brand relationships. In addition, this framework provides practical implications for retail environment design and customer-brand relationship management.

## Introduction

In the retail industry, a format blurring phenomenon has been observed, where consumers perceive increasing similarity between retailers who sell the same merchandise carried by many of their competitors (Berman, 2010; Rudawska and Bilinska-Reformat, 2018). It has thus become particularly crucial to create unique impressions of a retailer brand among consumers. One important way to differentiate a retail brand from those of its competitors has been recognized as building a unique brand personality (e.g., Jara and ans Cliquet, 2012). However, despite the fact that a body of literature has recognized the advantages of applying brand personality in the retail domain, the way retailers' brand personalities affect customers' perceptions of retailers, which in turn influence increased positive word-of-mouth (WOM) and patronage intention, is still not well-understood. Researchers have also called for more work focusing on understanding how and under what circumstances this symbolic usage of a brand affects the decision-making of the shoppers (e.g., Mohan et al., 2012; Liu et al., 2017; Radler, 2018), accentuating the importance of investigating a framework of retail brand personality and associated consumer outcome behavior when situational factors are incorporated.

More specifically, based on brand personality research in the context of both products and retail stores, the influence of brand personality on various consumer choices and purchasing behavior has been examined using self-congruity theory (e.g., Liu et al., 2012; Lee et al., 2020). However, the findings suggest that while consumers prefer brands with a personality that is consistent with their own self-concepts (e.g., one's self-identity), research showing whether the congruence between self-concept and perceptions of the brand leads to consequences, such as brand attachment, brand choice, and consumer loyalty and preferences, is inconclusive (e.g., Liu et al., 2012; Sop and Kozak, 2019). The research premise that self-concept is invariant across situations has been suggested as one possible cause for these inconclusive findings. Identity theory, which posits that self-concept is multidimensional, varies across different social situations, and differs in specific private or public shopping situations (e.g., store visibility and co-shopping), has been suggested to influence self-concept thereby impacting the congruity of self-concept with brand personality (Sirgy et al., 2000; Sirgy, 2018). Many researchers investigating retailing have either not applied this notion of a dynamic self-concept to compare different types of self-concept congruence (e.g., private, public) or have not incorporated situational shopping factors into their research design (e.g., Liu et al., 2012; Sop and Kozak, 2019). Their findings have thus been mixed.

Further, based on the social identity theory (Tajfel and Turner, 1979), which asserts that people define their self-concepts by identifying themselves as members of specific social groups, the customer-brand identification (CBI) framework (Lam et al., 2010) demonstrates that customers can perceive, feel, and value their sense of belongingness with a brand, which in turn contributes to sustained consumption. Applying the notion of CBI to retail brands, consumers who perceive a close bond with a retail brand, defined as retail brand identification, should, therefore, have strong motivations to patronize the retailer. Based on suggestions by social identity theorists (Oakes, 1987), the precondition of group identification is a salient perception of membership that is related to the similarities shared between a group and its identifiers. It is likely that self-congruity with a brand personality causes consumers to develop a vivid perception of identification with that brand. Therefore, the current study suggests that brand personality may increase customers' intention to provide positive WOM communications about and to patronize a focal retailer through situational self-congruity, leading to retail brand identification.

To address the aforementioned research problems, the current work is aimed toward proposing a “Retail Brand Personality-Behavioral Outcomes” framework to test the relationships among consumer perceptions of a retailer's brand personality, the congruity between retail brand personality, and different types of consumer self-concept, consumers' identification with the retail brand, and outcome behaviors, such as patronage intention as well as positive WOM. Moreover, due to the unique nature of retail environments (i.e., stores), identification with a retail brand may be based on different cues relative to identification with a product brand. For example, retailers provide customers with observable clues (e.g., sales personnel, other shoppers, the appearance, and presentation of the merchandise) with which to evaluate “the brand group” and with opportunities to experience “being in the group.” The perceived identification with a retail brand is likely to be more specific than with a product brand (i.e., CBI). Therefore, another purpose of this study is to conceptualize “Retail Brand Identification” (RBI), which specifically describes the perceived bond between a retail brand and shoppers.

This study investigated two fundamental research questions. First, the current study attempts to determine how self-congruity with retail brand personality is influenced by various social situational factors (e.g., shopping alone, co-shopping, high store visibility, etc.). The second research question addresses how perceptions of retail brand personality contribute to positive WOM communications and increased patronage intention toward a retailer through consumers' identification with the retail brand (i.e., RBI).

## Literature review and hypothesis development

### Retail brand personality

Retail literature has long recognized that consumers have no problems with imbuing retail brands with human-like characteristics (Merrilees and Miller, 2001; Kim et al., 2018) and that brand personality can serve as an effective means of retailer differentiation and positioning against competitors (Kim et al., 2015). However, research investigating the brand personality of retailers is relatively limited compared to the large body of literature on brand personality in general. The concept of retail brand personality was first brought to attention by Martineau (1958) but was formally defined, 60 years later, by Das et al. (2012a) (p. 98) as “a consumer's perception of the human personality traits attributed to a retailer's brand.”

Studies in the retail branding literature have attempted to identify the dimensions of retail brand personality. Although, Aaker (1997) developed the well-known brand personality scale (BPS) and identified five dimensions for all brands, a stream of research has been arguing that the dimensions of BPS cannot delineate those of retail brands because retail stores are multisensory in nature (Ailawadi and Keller, 2004; Das et al., 2012a), which makes their brands distinct from product brands. Therefore, some researchers have developed specific scales to describe unique dimensions of brand personality in various retail formats. For e.g., Helgeson and Supphellen (2004) identified *Modern* and *Classic* as dimensions for fashion retailer brands. While others, like Das et al. (2012b), identified *Sophistication, Dependability, Empathy, Authenticity*, and *Vibrancy* as dimensions for department stores.

Based on empirical evidence, a favorable, distinct retail brand personality exerts significant influences on various consumer behavior. For example, certain traits of retail brand personality (e.g., autonomy, sincerity, conscientiousness, etc.) were observed to be positively associated with brand trust (Lombart and Louis, 2012), brand loyalty (Das et al., 2012b; Das, 2014; Lombart and Louis, 2014), brand preference (Maehle and Shneor, 2010), and patronage intention (Rahman et al., 2016). This symbolic usage of retail brands was also found to enhance brand love (Roy et al., 2016), which is defined as a strong affection for a brand. Despite these empirical studies showing a positive relationship between retail brand personality and consumer behavior, exceptions were also found. For instance, Lombart and Louis (2016) found the traits of retailer personality (e.g., conscientiousness and agreeableness) did not influence loyalty. Roy et al. (2016) also suggested that the personality of store brands (i.e., excitement and sincerity) did not predict positive WOM. These exceptions underscore the insufficient understanding of retail brand personality and the need for more investigations into its influences as well as the underlying mechanisms (i.e., mediating and moderating factors).

### Self-motives: Self-verification and self-enhancement

Self-concept is a social product (e.g., Rogers, 1959). After formation, self-concept is continuously verified and enhanced *via* feedback from social interactions throughout people's lives (Swann, 1983) and thus can exert potent influences on consumption behavior (Anand and Kaur, 2018). The theory of self-verification, developed by Swann (1983); Swann and Brown (1990), postulates that people have needs to verify, validate, and sustain their existing self-concepts. Based on the study by Swann et al. (1989), people tend to seek subjectively accurate social feedback, whether positive or negative, that is consistent with their self-concept for self-verification purposes. However, it is also suggested that the self-views held by psychologically-healthy people are usually biased in a positive direction (Escalas and Bettman, 2003). Compared with negative characteristics, positive personality information is easier to process and recall (Kuiper and Derry, 1982). With positive self-evaluations, most people tend to prefer and seek positive over negative social feedback (Giesler et al., 1996). From a marketing perspective, the literature has documented that self-verification can be accomplished by consuming a good (e.g., Yang et al., 2018), a brand (Malär et al., 2011; van der Westhuizen, 2018), or by patronizing a specific restaurant (Stuppy et al., 2020) with an image or personality that is congruent with one's actual self. This can, in turn, lead to positive self-evaluations (Burke and Stets, 1999).

Whereas, self-verification focuses on a need to obtain feedback from others that confirms one's self-identity, self-enhancement (Jones, 1973; Shrauger, 1975) emphasizes people's desires for favorable and/or “better” self-concepts in order to enhance their self-esteem (Higgins, 1987). People strive to create good impressions in order to gain social approval and to obtain the intrinsic satisfaction derived from presenting an ideal self-image (Schlenker, 1980). To achieve self-enhancement, people are likely to affiliate with a group that reflects their desired identity (Frischlich et al., 2014). They can also consume a product (Makkar and Yap, 2018) or brand (Malär et al., 2011) or donate to a specific organization (Zogaj et al., 2021) with an image or a personality that reflects the person's aspirations and dreams.

### Situational self-congruity

The main tenant of self-congruity theory is that customers prefer a brand corresponding to their self-concept, which has been considered to have an active, multifaceted structure and to reside in the social structure (Markus and Wurf, 1987; Anand and Kaur, 2018). Using the premise of a dynamic self-concept, situational congruity is thought to serve as a mechanism by which each self and its related sets of personality traits are accessed through social situations. It is thus likely that consumers visit different retail stores with different self-concepts (e.g., the actual or ideal self) and under different conditions; and that they prefer retail brands consistent with the particular self-concept that surfaces in a specific situation. However, the literature applying dynamic self-concepts and situational self-congruity in retail contexts is rather limited. While some studies still presume an invariant self-concept across situations and find self-congruity to be a weak predictor of consumer behavior (Yu et al., 2013; Das, 2015; El Hedhli et al., 2016), others compare the influences of different types of self-congruity (e.g., actual vs. ideal self-congruity) on store choices without including the situational factors behind the congruencies in self-concept. Consequently, these studies have produced mixed results (Sirgy and Samli, 1985; Ekinci and Riley, 2003; Malär et al., 2011).

Based on previous empirical work, Sirgy et al. (2000) offered a conceptual model of the retail environment, self-congruity, and patronage that proposed that each type of self-congruity among retail brands can predict patronage through specific types of self-motivation (i.e., self-consistency and self-enhancement) that are influenced by situational factors. They referred to actual and ideal self-congruities as “private self-type self-congruities,” and social and ideal social self-congruities as “public self-type self-congruities” (p. 132–133). This is the framework of self-congruity adopted in this research model.

In fact, Sirgy et al. (2000) also proposed four situational factors that stimulate different forms of self-congruity: store conspicuousness (the perceived likelihood that shoppers might be seen by significant others while shopping), co-shopping (motivation to gain social approval), shopper's age, and response mode (i.e., whether the decision is about a preference or a brand choice). Each of these situational factors was proposed to influence the ability of the four self-congruities to predict retailer patronage through influencing the correspondent dimension of self-concept. However, while these situational factors are logically appealing and worthy of further research, they have not been empirically tested in terms of predicting behavioral outcomes.

### Retail brand personality and self-congruity

Due to the characteristics of the internal environments of retailers, retail brand personality can include a negative dimension with a set of correspondent negative traits (d'Astous and Lévesque, 2003). However, according to the self-verification (Giesler et al., 1996) and self-enhancement theories (Swann, 1983), consumers may only relate to the positive dimensions and personality traits of brands. The self-verification theory postulates that people need to verify their existing self-concepts (i.e., actual and/or social selves) by seeking experiences that sustain their sense-of-self, which is usually biased in a positive direction among psychologically-healthy people (Escalas and Bettman, 2003). Therefore, by applying the self-verification theory, it is likely that people only relate positive retail brand personalities to their actual and/or social selves. Self-enhancement theory predicts that people have a tendency to search for experiences that can improve their feelings and self-values (Sedikides and Strube, 1997), thereby enhancing their self-esteem (Higgins, 1987). Thus, it is likely that people only associate attractive or positive retail brand personalities with their ideal and ideal social selves. Applying both self-verification and self-enhancement theories, it is hypothesized that consumers only associate the positive personalities of a retail brand with their own personalities. This assumption leads to Hypothesis 1.

*H1: Positive perceptions of a retail brand personality lead to a higher (a) private self-congruity and (b) public self-congruity*.

### Retail brand identification

#### Theoretical background of RBI: Social identity theory and its extension

Researchers have long discussed that people can identify themselves as members of an organization (Lee et al., 2015), a company (Bhattacharya and Sen, 2003), or a brand group (Lam et al., 2010), regardless of whether they have a formal membership (e.g., Pratt, 1998) or contact with other specific members (Turner, 1982). This discussion stems from the social identity theory (Tajfel and Turner, 1979), which posits that people can define their self-concepts by identifying or categorizing themselves as members of specific social groups. This socialization identification takes place when individuals perceive oneness and share a joint identification with a group of people (Ashforth and Mael, 1989).

Based on the social identity theory, Lam et al. (2010) proposed CBI, which describes the bonds customers have with a brand. Based on Lam et al. (2010), brands are a type of social and symbolic entity with which customers are able to relate and identify. The CBI is formally defined as “a customer's psychological state of perceiving, feeling, and valuing his or her belongingness with a brand” (Lam et al., 2010, p. 129). The “belongingness” in CBI refers to “psychological oneness” with the brand and stems from perceptions of group membership (p. 129).

#### Conceptualization of RBI

Due to their direct and continuous interactions with the consumer, the nature of retail businesses is very different from that of manufacturing businesses. All the store environment-related consumer interactions can influence consumers' perceptions of a retailer's symbolic characteristics (i.e., its brand image). Retail brand personality has been shown to be more comprehensive than brand personality, in general, and includes negative characteristics that brand personality does not have (d'Astous and Lévesque, 2003). Using the same logic, it is argued that in identifying with a retail brand, consumers may employ different cues relative to those used when identifying with a product brand. In other words, the nature of RBI may differ from that of product brand identification. Therefore, this study extends the notion of CBI (Lam et al., 2010), which focuses on product brands, to RBI. The RBI is defined as “a customer's knowledge (cognitive identification) that he/she belongs to a retail brand together with perceived affect toward (affective identification) and perceived value (evaluative identification) of the membership.” The RBI takes place when customers recognize the oneness, they share with the retail brand and feel good and proud of their relationship with the brand.

Regarding the environmental factors that affect consumer perceptions of retail brands, two reasons can justify why RBI may be perceived as more complex than CBI in the minds of retail customers. First, store employees, such as sales personnel, provide a visible reference group for identification, which is usually absent in product brand identification. In stores, sales personnel can both be directly observed by and can interact with shoppers. They are social cues (Kumar and Kim, 2014) or symbols of the retail brand (Davies and Chun, 2012), communicating the brand values to customers.

Second, the physical store *per se* provides a place or an opportunity for “being in the group,” which makes RBI less symbolic than product brand identification. The literature reports that elements of a store environment, such as ambient designs, produce emotional effects on consumers (Koo and Kim, 2013) and influence how they value their shopping experiences (Bonnin and Goudey, 2012), providing clear clues about how being in a group feels and looks. A store environment even provides an opportunity to meet and interact with the “in-group” members (i.e., other shoppers and store employees), thereby making the group more tangible. In other words, a store environment creates a “group location,” facilitating or triggering customer identification with the store brand. When actually in a store, customers are able to experience and confirm their “belongingness” with the store brand. As a result of the store experience, the identification with retail brands may become less symbolic than with product brands, which typically lack observable and/or reachable group references and opportunities for confirmation.

#### Self-congruity and RBI

Social identity theory proposes that people tend to categorize themselves into various groups, such as members of sporting clubs or fans of TV shows (Trepte, 2006). The perception of having group membership has to be salient to positively influence the corresponding social identity (Oakes, 1987) and initiate a behavior consistent with others in the group (Tajfel, 1979; Hogg and Reid, 2006). The salience described using social identity theory is the “psychological significance of a social membership,” (Stets and Burke, 2000, p. 230) which is termed “psychological salience” (Oakes, 1987). The occurrence of psychological salience depends on whether two determining factors, “accessibility” and “fit,” are present (Oakes, 1987). Accessibility refers to the “readiness” of a social category to gain salience (Oakes, 1987, p. 127). Greater readiness contributes to less effort needed for identification. Certain aspects of self-concept (e.g., gender and race), which are self-evident and chronically accessible in the mind, have been considered to be both situationally and chronically salient (Hogg and Reid, 2006). Since personality is part of one's self-concept, a congruent retail brand personality should be recognized by customers as being accessible, thereby becoming salient enough to influence social (retail brand) identification.

The other determinant of psychological salience, “fit,” refers to the degree to which there are similarities and differences observed between people and respective social groups (Oakes et al., 1991). The more similarities that are observed, the greater the likelihood that the social group will be credited as being optimally fit. As such, the perception of personality congruence with a retail brand, which captures similarities between customers and the retail brand, is likely to promote the retail brand as having a good fit. Under the assumption that a congruent retail brand personality has both accessibility and a good fit for customers, the corresponding brand is likely to be psychologically salient; and will therefore positively influence RBI. In other words, the level of self-congruity with the retail brand personality may be an antecedent of RBI. Therefore, Hypotheses 2a and 2b are stated as follows:

*H2: The stronger the congruence in the case of both (a) private self-concepts and (b) public self-concepts with retail brand personality, the stronger the perception of overall identification with the retail brand*.

#### Retail identification, WOM, and patronage intention

The social identity theory suggests that people display group behavior that includes in-group favoritism and out-group discrimination as a part of their social identity processes (Tajfel, 1979; Trepte, 2006). Tajfel (1979) devised an experiment and showed that simple categorization of a person into a group is sufficient to make people favor their in-group and discriminate against the designated out-group. Some laboratory studies have also demonstrated that in-group favoritism can occur under conditions in which the categorizations are random and group memberships are anonymous (Billig and Tajfel, 1973).

In marketing contexts, in-group favoritism has been translated into the commitment to achieve the goals of companies in the form of positive WOM (Kim et al., 2001; Brown et al., 2005) and purchasing (Lam et al., 2010; Maison and Maliszewski, 2016). Applying this logic, overall RBI is likely to be associated with providing positive WOM about a retail brand and displaying high levels of retailer patronage intention. In addition, each of the dimensions of overall RBI (i.e., cognitive, affective, and evaluative) are also likely to be associated with providing positive WOM about a retail brand and displaying high levels of retail patronage intention. Therefore, hypotheses 3 and 4 posit the following relationships:

*H3: The stronger the (a) overall RBI, (b) cognitive RBI, (c) affective RBI, and (d) evaluative RBI, the greater the intention to convey positive WOM communications about the retailer*.

*H4: The stronger the (a) overall RBI, (b) cognitive RBI, (c) affective RBI, and (d) evaluative RBI, the greater the intention to patronize the retailer*.

#### The moderating effect of the shopping situation

Recognizing the nature of situational self-congruity, Sirgy et al. (2000) proposed four factors (i.e., store visibility, co-shopping, age, and response mode) that may influence shoppers' self-concept dimensions and, consequently, impact self-congruity and retail patronage. The first factor, store visibility, refers to the perceived likelihood that shoppers might be seen in a store by their significant others (i.e., friends, relatives, etc.) (Sirgy et al., 2000). For example, a store located in a crowded public place, such as a shopping mall or on a busy thoroughfare, is considered as being more visible than a store located on a quiet, secluded street. High store visibility is likely to influence one's public self-congruity, whereas low store visibility is likely to influence one's private self-congruity. Co-shopping, the second factor, is related to the motivation for gaining social approval (Sirgy et al., 2000). Thus, co-shopping with others is likely to be a cue that positively influences the relationship between public self-congruity and RBI, whereas shopping alone is likely to be a cue that positively influences the relationship between private self-congruity and RBI. The third factor, age, influences the dominance of self-concept dimensions. Younger people are more preoccupied with making good impressions on others and are more likely to have a stronger sense of public self than older consumers. The fourth factor, response mode, refers to “whether the decision is a preference judgment type or a brand choice” (Sirgy et al., 2000, p. 134). A preference judgment-type decision is likely to positively influence self-esteem, which is related to one's ideal self, whereas a choice-type decision is likely to positively influence self-consistency, which is associated with one's actual self (Sirgy et al., 2000). Compared with store visibility and co-shopping, which serve as external cues influencing shoppers' perceptions of self from a retailer's environment, the other two factors, age and response mode, serve as internal factors. This study focuses on situational factors that retailers can manage in order to strengthen the relationships among both types of self-congruity and retail brand identification. Therefore, only the more manageable external cues were included in this research.

Since the external factors, store visibility and co-shopping, are both related to whether a shopper might be seen by significant others, in this study, they are combined into one factor, *shopping conspicuousness*, which is conceptualized as the visibility of the shopping behavior to significant others (e.g., the shoppers' friends, family, important social cohorts, etc.). A situation high in shopping conspicuousness comprises high store visibility and co-shopping, whereas one low in shopping conspicuousness encompasses low store visibility and shopping alone. Based on the conceptual model proposed by Sirgy et al. (2000), public-type self-congruity should be more influential than private-type self-congruity in predicting retail patronage under the conditions of high store visibility and co-shopping. Therefore, Hypotheses 5a and 5b are stated as follows:

*H5a: In shopping situations regarded as being low in shopping conspicuousness, private self-congruities will more strongly impact the overall RBI than social public self-congruities*.

*H5b: In shopping situations regarded as being high in shopping conspicuousness, social public self-congruities will more strongly impact the overall RBI than private self-congruities*.

## Methods

### Research design

To test the proposed model relationships, data were collected using a survey with descriptions of two retailer formats that depicted a retailer image format of either a department store or a discount retailer as the context. These two formats were selected because most consumers are familiar with them and, therefore, will have exemplars for each type (Keaveney and Hunt, 1992). It also varied the conspicuousness of the shopping situation (i.e., high/low shopping conspicuousness). Therefore, this study used a 2 (retailer image format) x 2 (shopping situation conspicuousness) between-subjects mixed design, in which the subjects were randomly assigned to one of the four groups who read a scenario that provided a description of a retailer format of either a hypothetical department (*n* = 311) or discount store (*n* = 305), and a description that presented a high (*n* = 303) or low (*n* = 313) context of shopping conspicuousness. On exposure to a scenario appropriate for each particular condition, the subjects then completed the survey conducted *via* a self-administered online questionnaire that employed the questions measuring the variables in the proposed research model.

### Pre-test

The scenarios used in the survey provided information for determining the perceptions of the retail brand personality associated with two different retailer image formats (i.e., either an upscale department store retailer or a discount store retailer) and assessing the influence of situational cues (high or low shopping conspicuousness) on the respective relationships between participants' private or public self-congruities and their degree of RBI. These scenarios were first refined *via* two focus groups of college employees (10 in total) from a north-central university in the United States to confirm whether exposure to each scenario produced the desired results. Further, two pretests were conducted using two convenience samples of 123 and 91 undergraduate students from both the aforementioned university and a university in the southwest of the United States, respectively, to further test each scenario and affirm whether the questionnaire instructions and format were easy to understand.

### Manipulation checks

Subjects in the pre-tests and the main study also responded to several measures developed by the researchers for manipulation checks and verified that the manipulations of both the retailer image format and the shopping conspicuousness situations were perceived as intended. Based on the results from the main data collection, the hypothetical department store description was rated as being more similar to the well-known department store retailer exemplars (Macy's, Dillard's, Nordstrom; *M* = 5.11) and less similar to the well-known discount store retailer exemplars (i.e., Wal-Mart, Target, Sears; *M* = 3.69) (*p*'s ≤ 0.05), whereas the hypothetical discount store description was perceived as being more similar to the discount store retailer exemplars (*M*= 4.78) than the department store retailer exemplars (*M* = 3.32) (*p*'s ≤ 0.05), supporting the premise that each store description produced the intended perceptions of department and discount store retailer image formats. For the shopping conspicuousness manipulations, the means of the six visibility perception items reported by subjects assigned to the high conspicuousness manipulation (*M* = 4.72) were significantly higher than those reported by subjects assigned to the low conspicuousness manipulation (*M* = 3.98) (*p*'s ≤ 0.05), supporting the supposition that each description of shopping conspicuousness situations was perceived as intended.

### Main study sample

The respondents were recruited through Qualtrics, an online survey research firm. The survey was administered to a sample of consumers in the United States who had made at least one purchase in the previous 6-months period from a department store and a discount store. A total of 627 responses were collected, of which 11 cases were identified as invalid, resulting in a total sample size of 616. The majority of the respondents were Caucasian (82.8%), above the age of 35 (79.1%), married (55.7%), and had at least completed high school (96%). There were slightly more females (58.8%) than males. In addition, almost half of the respondents (48.4%) had an annual household income of US$50,000 or more.

### Measures

#### Retail brand personality

Considering that the retail literature has not arrived at a consensus on the dimensions of retail brand personality, in this research, the retail brand personality scale from Aaker's (1997) BPS and the retail brand personality scales, developed for specific retail formats, by Darden and Babin (1994), d'Astous and Lévesque (2003), and Helgeson and Supphellen (2004) were used. The scale adaptation prior to the main study was implemented in three major steps: First, an integrated framework of retail brand personality dimensions, comprised of similar traits from scales developed in the aforementioned literature, was identified. Second, a focus group, composed of seven graduate students (three Caucasians, two African-Americans, one Latino, and one Asian), was used to assess the relevance of these dimensions and the traits making up each dimension to the retailing context in order to provide insights for deleting and/or developing additional adjectives that could be employed to describe the personality traits of retailers.

Finally, to refine the scale, testing was conducted on a convenience sample of 297 undergraduate students (197 females, 97 males, and 3 not identified). Each participant was randomly assigned to a survey that used either Walmart or Macy's as the retailer context (Walmart: 148 subjects; Macy's: 149 subjects). The participants were asked to rate, on a 7-point scale ranging from 1 (least descriptive) to 7 (very descriptive), the extent to which they felt each of the characteristics provided in the preliminary list may be descriptive of the retailer identified in the survey (i.e., either Walmart or Macy's). An exploratory factor analysis was used to identify the items that would be employed in the retail brand personality scale for this research. The results of the exploratory factor analysis suggested a five-dimension scale (a lower number of dimensions than the conceptual scale) with 48 items (loading ≥ 0.40) retained. The five dimensions were labeled: Sophisticated, Genuine, Inactive, Solidity, and Ruggedness (% variable extracted: 4.09–25.66; Cronbach's α: 0.68–0.97). For simplicity, only the three traits in each factor with the highest factor loadings were included in the main survey. In the main study, only three factors were retained for the structural equation model (% variable extracted: 14.86–28.23; Cronbach's α: 0.85–0.90): *Inactive, Genuine, and Sophisticated*; thus, the model respectively consisted of six, six, and three items.

#### Self-congruity

The two-step measure of self-congruity used by Malär et al. (2011) was adopted, which directly evaluates the degree of congruity. First, the participants were instructed to think about the hypothetical store as a person and assign it the most descriptive characteristics (i.e., assess retail brand personality traits). The participants were then asked to think about their own personalities and indicate, on a 7-point Likert scale from 1 (strongly disagree) to 7 (strongly agree), the extent to which the personality of the hypothetical retailer matched their private and public self-concepts, using four items for each self-concept (e.g., “The personality of this store is a mirror image of how other people see me.”). In the preliminary exploratory factor analysis (EFA), the items were loaded on two factors (% variable extracted: 14.05–62.46); each consisting of four items intended to measure four dimensions of self-congruity (i.e., actual, ideal, social, and ideal social self-congruity), respectively. To be consistent with the theoretical framework, all of these items were further grouped into two separate a priori scales to measure *private* (i.e., actual and ideal) and *public* (i.e., social and ideal social) *self-congruity*. Each of the *private* and *public self-congruity* constructs had an adequate level of scale reliability (i.e., the Cronbach's α ranged from 0.824 to 0.848) and was used in further data analyses.

#### Retail brand identification

Retail brand identification was measured by using six items adapted from the CBI measure (Lam et al., 2010). For the cognitive identification dimension, the degree of overlap between customer identity and retailer identity was measured with two semantic items, one of which was adapted from the Venn diagram into a Likert scaled item, and the other was the verbal item originally used to cross-validate the Venn diagram (e.g., “I feel that I would share a common identity with this store”). The third and fourth items, which were aimed at measuring affective identification, and the fifth and sixth items, which were aimed at measuring the evaluative dimension, were adapted to reflect the retailer setting of this study. For example, the fourth item was changed from “stop using a brand” to “stop shopping in the hypothetical department store” (e.g., “I would feel disappointed if I had to stop shopping in this store.”). In the preliminary EFA, two factors were extracted (% variable extracted: 18.33–42.65; Cronbach's α: 0.66–0.67), which were considered to be acceptable based on the criteria (Cronbach's α > 0.60) suggested by Griethuijsen et al. (2015). Each factor consisted of three items intended to assess the three dimensions of the RBI (i.e., Cognitive, Affective, And Valuable *RBI*), respectively. Only the first factor, having the higher percentage of variance extracted, was retained, and treated as a unidimensional overall RBI construct in the further confirmatory factor analysis (CFA).

#### Outcome behaviors

In this research, positive WOM was measured using three items (e.g., “I would recommend this store to my friends.”) adapted from Maxham III (2001) and measured on a 7-point Likert scale ranging from 1 (e.g., very unlikely) to 7 (e.g., very likely). One item (loading <0.4) was excluded, and two items were retained for further CFA. (% variable extracted: 61.96; Cronbach's α: 0.92). Patronage intention was assessed with four items (e.g., “How likely would you be to patronize the hypothetical retail store in the future?”), which were adapted from Dabholkar and Baggozzi (2002), and measured on a 7-point Likert scale from 1 (very unlikely) to 7 (very likely). All the items were loaded on a single construct (% variable extracted: 80.67; Cronbach's α: 0.92) in the preliminary EFA.

## Data analysis and results

### Confirmatory factor analysis

Based on the results of the EFA, the retained items were further tested using a CFA to build the measurement model. For the retail brand personality constructs, a total of six indicators were excluded due to the strong correlations with other such constructs, leaving three items (tired, lazy, and sluggish) intended to measure the *Inactive* personality dimension, four items (Reliable, Reputable, Thriving, and Sincere) intended to measure the *Genuine* dimension, and two items (Dressy and Elegant) intended to measure the *Sophisticated* dimension. For the self-congruity constructs, due to the multicollinearity issues between *Private* and *Public Self-congruity* (e.g., *r* > 0.9), two indicators of each construct were excluded. Two retained items served as indicators of social and ideal social self-congruity (i.e., *Public Self-congruity*), and the other two retained items served as indicators of actual and ideal self-congruity (i.e., *Private Self-congruity*). Each dimension of self-congruity demonstrated appropriate content validity.

For *RBI*, the indicator intended to measure cognitive RBI had a low factor loading (i.e., 0.43), but was retained in order to maintain proper content validity. The CFA supported the *Patronage Intention* and *Positive WOM* factor structure identified by the EFA.

Table 1 shows the final measurement model results. This final measurement model fit the data well (*X*^2^ = 459.75 (*df* = 179; *p* ≤ 0.001); RMSEA = 0.051; CFI = 0.981; NFI = 0.970; GFI = 0.936; AGFI = 0.910), and the factor loadings were significant (*p* < 0.01). According to Hair et al. (2010), the convergent validity of latent constructs can be assessed by evaluating the individual standardized factor loading and by computing the composite reliability and extracted variance of each construct. Except for RBI, all constructs had substantial factor loadings (0.67–0.95), and every construct, including RBI, had composite reliability above 0.9 and an AVE above 60%, thereby suggesting adequate convergent validity.

**Table 1:** Measurement model results.

	**Std. loading**	***t*** **Value**	**SE**	**CR**	**AVE**
ξ_1_ *Sophisticated*				0.97	86.2%
χ_1_ Dressy	0.815	19.170	0.042		
χ_2_ Elegant	0.977	22.509	0.043		
*ξ_2_ Genuine*				0.98	71.4%
χ_3_ Sincere	0.787	21.847	0.036		
χ_4_ Reputable	0.770	21.200	0.036		
χ_5_ Reliable	0.758	20.741	0.037		
χ_6_ Thriving	0.672	17.658	0.038		
ξ_3_ *Inactive*				0.98	77.9%
χ_7_ Tired	0.718	19.371	0.037		
χ_8_ Lazy	0.810	22.560	0.036		
χ_9_ Sluggish	0.893	25.701	0.035		
η_1_ *Private Self-congruity*				0.98	82.7%
γ_1_ … a mirror image of how I “actually” see myself	0.837	23.229	0.035		
γ_2_ … consistent with how I would “ideally” like to see myself as being	0.886	24.963	0.038		
η_2_ *Public Self-congruity*				0.97	76.2%
γ_3_ … a mirror image of how other people see me	0.776	20.600	0.038		
γ_4_ … consistent with how I would like other people to think about me	0.819	21.929	0.037		
η_3_ *RBI*				0.97	62.8%
γ_8_ I would feel that my sense of who I am overlaps with my sense of what the retail store represents	0.429	9.888	0.043		
γ_9_ If someone praises this store, I would gladly join the conversation	0.731	18.161	0.040		
γ_10_ I would consider myself to be a valuable customer of this store	0.745	18.541	0.040		
η_4_ *WOM*				0.98	89.6%
γ_11_ How likely would you be to spread positive communications about this store	0.906	28.398	0.032		
γ_12_ I would recommend this store to my friends	0.947	30.494	0.031		
η_5_ *Patronage Intention*				0.99	81.8%
γ_13_ How likely would you be to patronize this store in the future …	0.801	23.515	0.034		
γ_14_ How certain are you that you might choose this store for your future shopping	0.912	28.832	0.032		
γ_15_ …the probability that you might choose this store for your future searching and purchasing activities	0.907	28.561	0.032		
γ_16_ …the extent to which you think that you might patronize this store in the future	0.760	21.396	0.036		

### Structural model

**Figure 1:**
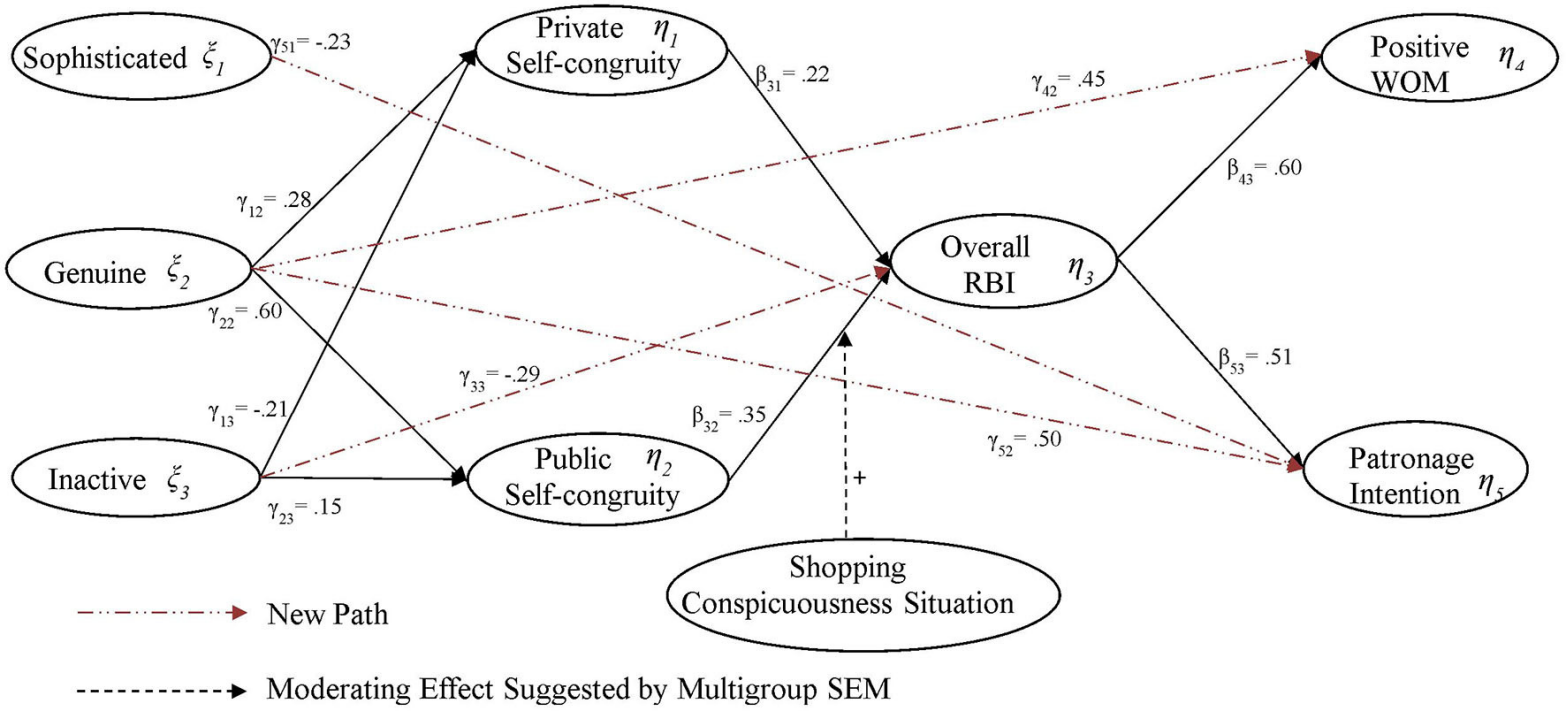
Retailer brand personality-behavioral outcomes final structural model for department and discount retailer image formats.

Structural equation modeling was run on the final theoretical model that consisted of three exogenous constructs (i.e., *Inactive, Genuine*, and *Sophisticated*) and five endogenous constructs (i.e., *Private Self-congruity, Public Self-congruity, RBI, Patronage intention*, and *Positive WOM*). The results showed that the theoretical model fit the data modestly well: chi-square of 720.54 (*df* = 192; *p* ≤ 0.001); CFI = 0.97; NFI = 0.96; GFI = 0.92; AGFI = 0.89; RMSEA = 0.06; *x*^2^/*df* = 3.75. However, two non-significant paths and modification indices suggested that a better model fit should be explored. The revised model (see Figure 1) fit the data better: chi-square: 502.57 (*df* = 190; *p* ≤ 0.001); CFI = 0.98; NFI = 0.97; GFI = 0.93; AGFI = 0.91; RMSEA = 0.05; *x*^2^/*df* = 2.65.

#### Retail brand personality dimensions

As predicted, *Genuine*, the positive perception of a retail brand personality, led to higher perceptions of both *Private* (γ_12_ = 0.28) and *Public Self-congruity* (γ_22_ = 0.60), thereby supporting H1a and b. However, the other positive dimension of retail brand personality, *Sophisticated*, had no effects on either *Private* and *Public Self-congruity*, which did not support H1a and b. The negative dimension of retail brand personality, *Inactive*, was observed to exert negative effects on *Private Self-congruity* (γ_13_ = −0.21), which supported H1a; it also exerted a positive effect on *Public Self-congruity* (γ_23_ = 0.15), which was contrary to H1b. While, Giesler et al. (1996) contended that most people tend to seek positive over negative social feedback, this finding seemed to support the experimental results reported by Swann et al. (1989), who found that, for self-verification purposes, people may seek negative social feedback if this is consistent with their self-concept (i.e., actual and social self). It is likely that *Inactive* was positively associated with social self-congruity for the purpose of verification, thus increasing the perception of *Public Self-congruity*. To test this assumption, several linear regression analyses were conducted, with the four types of self-congruity serving as dependent variables and the three dimensions of retail brand personality serving as independent variables. The results confirmed that *Inactive* had a positive association only with social self-congruity (β = 0.164^**^). Even though perceived *Inactive* contributed to social self-congruity, thus increasing perceived *Public Self-congruity* (γ_23_= 0.15), it exerted a stronger negative effect on *Private Self-congruity* (γ_13_= −0.21) as well as on the *overall* RBI (γ_33_= −0.29). Therefore, the total effect of perceived *Inactive* on the *overall RBI* was still negative (γ_23_ + γ_13_ + γ_33_ = −0.35). Even so, the mixed results regarding the relationships among the three dimensions of retail brand personality and the two types of situational self-congruity demonstrated partial support for H1a and b.

#### Self-congruities and RBI

The results also revealed that both *Private* and *Public Self-congruities* were positively related to the overall *RBI* (β_31_= 0.22; β_32_= 0.35), supporting H2a and b. The results also showed that, in turn, the stronger the overall *RBI*, the greater the intention to convey positive *WOM* about the retailer (β_43_= 0.60) and to patronize the retailer (β_53_= 0.51), thereby supporting H3a and H4a, respectively. To test the relationships between each of the *RBI* dimensions and these behavioral outcomes, two separate linear regressions were conducted with *WOM* or *Patronage Intentions* as the dependent variable and the three dimensions of RBI (i.e., cognitive, affective, and evaluative) as the independent variables. *Cognitive* RBI was not related to either *WOM* or *Patronage Intentions*. Therefore, H3b and H4b were rejected. However, both *Affective RBI* and *Evaluative RBI* had significant and positive associations with *WOM* (β_*Affective*_ = 0.22^**^; β_*Evaluative*_ = 0.29^**^) and *Patronage Intentions* (β_*Affective*_ = 0.22^**^; β_*Evaluative*_ = 0.32^**^), supporting H3c, H3d, H4 c, and H4d, respectively.

#### Moderation effects

The moderation effect of *Shopping Conspicuousness Situation (H5a* and *b)* was examined by conducting a multigroup SEM, which was run on the final model using high and low *Shopping Conspicuousness* settings. The results showed a good global model fit for the combined setting (chi-square = 807.76 (*df* = 380); *p* ≤ 0.001; RMSEA = 0.042, CFI = 0.950) and a good model fit for each setting (High: chi-square = 372.01, (*df* = 191); *p* ≤ 0.001, RMSEA = 0.056, CFI = 0.953; Low: chi-square = 379.97, (*df* = 191); *p* ≤ 0.001, RMSEA = 0.064, CFI = 0.947). The change of CFI between the two settings is −0.006, which is lower than −0.01, the cutoff suggested by Cheung and Rensvold (2002); this suggests measurement invariance was attained. The results also showed that the shopping conspicuousness situation did not moderate the *Private Self-congruity–RBI* relationship [Δ*x*^2^(1) = 2.68; H5a was rejected] but the *Public-Self-congruity–RBI* relationship [Δ*x*^2^(1) = 5.58; H5b was supported]. The *Public Self-congruity*–*RBI* relationship was greater under the high (β_3, 2_ = 0.687, *p* ≤ 0.05) than it was under the low (β_3, 2_ = 0.193, *p* ≤ 0.05) shopping conspicuousness situation.

A multigroup SEM was also run on the final model using the department and discount store formats to determine whether the proposed research model applied across retail formats. The results showed a good global model fit for the combined format (chi-square = 731.24 (*df* = 380); CFI = 0.98; NFI = 0.95; RMSEA = 0.055) and good model fit for each retailer format (Department Store Format: contribution to chi-square = 382.45, RMR = 0.06, GFI = 0.90; Discount Store Format: contribution to chi-square = 368.29, RMR = 0.06, GFI = 0.91), demonstrating that the format models fit the data well. The results of model fit comparisons revealed that most of the parameter estimates in the two retailer format models were consistent with those in the combined model. The major exception was that the path from *Private Self-congruity* to RBI became non-significant in the discount store format model (β_3, 2_ = 0.061; Δ*x*^2^ = 4.22).

To determine whether the moderating role of *Shopping Conspicuousness Situation* changes across different formats, two linear regressions with three-way interactions among *Retailer Image Format, Shopping Conspicuousness Situation*, and either *Private* or *Public Self-congruity* were conducted. The results showed that none of the three-way interactions were significant [Change in F (p) = 0.52(0.47), 0.09(0.76)], suggesting that the manner in which the *Shopping Conspicuousness Situation* moderated the relationships between *Private Self-congruity* and RBI and between *Public Self-congruity* and RBI did not change across the department and discount store formats.

Finally, in order to determine the common method variance (CMV), a Harman's (1967) one-factor test and an EFA were conducted on all items used in this study. The results of the principal component analysis with a varimax rotation revealed that there was no single factor in the factor structure accounting for a majority of the variance (CVE of the first factor: 27.26%), suggesting a low possibility that common method bias would undermine the results of this study.

## Discussion, conclusion, and limitations

The aim of this study was to empirically investigate the potential mediators and moderators among retail brand personality and the intentions to patronize and communicate positive WOM about a retailer. The results of this research provide four important contributions to the branding and retailing literature that have not been previously investigated. The first contribution was the integration of identity theory with social identity theory to: examine the relationships among consumer self-concepts as well as behavioral outcomes and reveal mediators in the relationship that had not been explored before. The second contribution of this study, previously conceptualized by Sirgy et al. (2000) but not empirically investigated, was the inclusion of social shopping situations to study self-congruity. This study provides evidence confirming that the influence of perceived self-congruity on consumers' behavior varies under different social shopping situational cues (i.e., the shopping conspicuousness situation). In addition, the third contribution was that this work is the first study to consider the unique nature of brand identification in retailing by examining RBI to provide an additional theoretical perspective for guiding future research on consumer relationships. Finally, the results of this study supplement recent research about the impact of retailer image formats on the relationships among retail brand personality and its outcome variables. The subsequent sections discuss the contributions of this study in accordance with the proposed objectives.

### The relationships between retail brand personality and self-congruity

The first objective of this study was to apply identity theory to examine the relationships between private and public self-congruity and retail brand personality under different social shopping situations. Based on self-verification and self-enhancement theories, it was hypothesized that positive perceptions of a retailer's brand personality would lead to a higher congruence between retail brand personality and private and public self-concepts, which was partially supported by the mixed results. Perceived *Genuineness*, the most influential positive dimension of retail brand personality, positively predicted both *Private* and *Public Self-congruity*. The other positive perception of retail brand personality, *Sophisticated*, had no effect on either *Private* or *Public Self-congruity*. It was reasoned that *Sophisticated* is more commonly associated with specialty stores than department or discount stores. For example, Zentes et al. (2008) investigated how BPS was applied in six different retail brands. They reported that Aldi (a German grocery retailer) was perceived as being strong on *Competence* and *Sinceri*ty personality dimensions but weak on *Sophistication*, whereas Douglas (the German market leader in perfumes and beauty care) was perceived as being strong on *Sophistication*. In addition, the *Sophisticated* items (i.e., Dressy and Elegant) might also be more descriptive of store brand personalities than the shoppers' own personalities because these items were not included in the Big Five personality scale used to measure human personality (Gosling et al., 2003).

The only negative dimension, *Inactive*, was observed to decrease the perceptions of *Private Self-congruity*, as predicted, consistent with Giesler et al. (1996), that most people tend to seek positive over negative social feedback. However, the results of this study also showed that *Inactive* positively predicts *Public Self-congruity*, rejecting H1b. Based on Swann et al. (1989), people look for accurate social feedback, positive or negative, in order to verify their actual and social self-concept, so it is possible that the significant path between *Inactive* and *Public Self-congruity* in this study is driven by a significant congruence between *Inactive* and social self-concept. This inference was justified by an additional set of linear regression analyses conducted by retail store format and over both combined store formats. This finding suggests that the positive association between the *Inactive* retail brand personality dimension and *Public Self-congruity* resulted from a positive association between *Inactive* and social self-congruity reflecting a purpose of self-verification. It is also likely that consumers perceive the inactiveness of an upscale department store retailer as congruent with their public selves because they still wish to be seen by others as purchasing merchandise from stores having an image of offering fashionable, higher-priced merchandise categories, and department stores have typically had that long-established image. It is important and interesting for future research to investigate how a retailer format might influence perceptions of a retailer's brand personality as well as the relationship between retail brand personality and consumers' perceived private and public self-congruities.

### The moderating role of the shopping conspicuousness situation

Applying the theory of situational self-congruity, it was hypothesized that the *Shopping Conspicuousness Situation* serves as a situational cue, changing the effects of *Private* and *Public Self-congruity* on perceived overall RBI. The results suggested that the *Shopping Conspicuousness Situation* moderated the relationship between *Public Self-congruity* and the overall RBI, but it had no effect on the relationship between *Private Self-congruity* and overall *RBI*. This result was applicable for both the department and discount store formats.

The finding that the relationship between *Public Self-congruity* and the overall RBI was stronger under a high rather than low *Shopping Conspicuousness Situation* was in agreement with the views proposed by Sirgy et al. (2000). When co-shopping with significant others in a highly visible retail shopping situation, the shoppers' public self-concepts are stronger than the other dimensions of self-concept, and the perceived congruence between retail brand personality and public self-concept becomes salient and influential, thereby strongly increasing the perception of the overall RBI. In contrast, when shopping alone in a relatively secluded retail shopping situation, shoppers' public self-concepts are not stronger than the other self-concept dimensions, and their perceived *Public Self-congruity*, which was not particularly salient, exerted a positive but relatively weak effect on perceptions of the overall RBI. Instead, the influence of *Private Self-congruity* on the overall RBI was slightly stronger than that of *Public Self-congruity* under the low *Shopping Conspicuousness Situation*. However, *Public Self-congruity* exerted a stronger influence on the overall *RBI* than it was in the case of *Private Self-congruity*, both when *Shopping Conspicuousness* was high and when the possible moderating effects of high and low shopping conspicuousness were not considered. These findings not only demonstrate that self-congruity, specifically the public-self type, is situational, but they also increase our understanding of the relationships between the different types of self-congruity and behavioral outcomes. The mixed results in the previous literature on how self-congruity predicts behavioral outcomes (He and Mukherjee, 2007; Malär et al., 2011) may be thus explained by the exclusion of situational factors in their investigations. The research premise of invariant self-concept across situations was proved to be inappropriate.

### The role of RBI

In this research, overall RBI was found to be a crucial mediator in the retail brand personality framework and its various outcome variables.

#### Retail brand personality, self-congruity, and RBI

The results supported the assumption that a congruent retail brand personality has both “accessibility” and “good fit” for customers, promoting the psychological salience of the perception of a correspondent retail brand membership that thus positively influences RBI. Both *Private* and *Public Self-congruity* positively predicted the overall RBI when the shopping conspicuousness situation and the retailer image format were not considered.

Regarding the relationships between retail brand personality dimensions and RBI, with the exception of *Sophisticated*, both the *Genuine* and *Inactive* dimensions influenced the overall RBI. For example, perceived *Genuineness* had a positive and indirect influence on the overall RBI through increased perceptions of both *Private* and *Public Self-congruity*. Relative to perceived *Genuineness*, perceived *Inactiveness* had a more complicated influence on the overall RBI. It had both direct and indirect influences that decreased the overall RBI. The indirect influence was comprised of a relatively weak positive influence on *Private Self-congruity* and a relatively strong negative influence on *Public Self-congruity*. It seems that even though consumers may perceive a small degree of congruence between their private selves and a negative brand personality, they do not feel a close bond with that brand.

#### RBI and behavioral outcomes

Consistent with the social identity theory, which posits that in-group favoritism is a part of the social identity process (Tajfel, 1979; Trepte, 2006), the results of the current research showed that the shoppers who identified with a retail brand were willing to favor the retailer by their intentions to convey positive *WOM* communications about and/or to patronize the retailer. The results also suggested that the positive influence of the overall RBI on outcome behaviors was driven only by *Affective RBI* and *Evaluative RBI*. This finding suggests that shoppers' perceived affect toward and value associated with brand membership have greater effects than the simple knowledge about membership with the brand, thus providing key elements to encourage in-group favoritism.

#### The role of RBI in the entire framework

Since neither *Private* nor *Public Self-Congruity* had direct associations with *WOM Communications* and *Patronage Intention*, the overall RBI fully mediated the relationships between the two types of self-congruity and the behavioral outcomes. This finding may help in understanding why self-congruity has sometimes been found to be a poor predictor of consumer behavior (Shank and Langmeyer, 1994; Ahn et al., 2013; Yu et al., 2013). Mere perceived similarities between consumers and brands may not be sufficient to directly increase outcome behavior like WOM and patronage intention. Rather, the influence of self-congruity on such outcome behavior should occur *via* initiating perceptions of a close bond between consumers and retail brands.

The mediating role of RBI on the relationships among retail brand personality dimensions and behavioral outcomes is also important. With the exception of perceptions that the brand was *Sophisticated, which* did not affect outcome behavior through either *Public* and *Private* self-congruity or overall RBI, the other two dimensions were found to have indirect effects on the outcome behavior through *Private/Public Self-congruities* and RBI together or RBI alone. *Inactive* had no direct effects on either *WOM* or *Patronage Intention*. That is, self-congruities together with the overall RBI fully mediated the relationship between *Inactive* and the behavioral outcomes in the model. Perceived *Genuineness* had both direct and indirect effects through self-congruities and the overall RBI on *WOM* and *Patronage Intention*. The crucial mediating role of RBI identified in this study may also explain why retail brand personality was sometimes found to have no effect on behavioral outcomes (Roy et al., 2016).

### Managerial implications

#### Retail brand personality

The measure of retail brand personality adapted for the purposes of this research revealed only three dimensions, which were less than the five dimensions in Aaker's (1997) BPS. Format blurring (Berman, 2010) may be a possible reason for consumers perceiving fewer differences in retail brand personality relative to product brand personality. The results of this research highlight the importance of establishing a retail brand personality that is perceived in consumers' minds as having positive dimensions, such as *Genuineness or Sincerity*. It is crucial for retail practitioners to understand how their target customers interpret their personalities as being aligned with a retail brand personality that exemplifies these positive personality dimensions. Based on previous research, the “price-quality ratio” may serve as a major determinant for each *Genuine* trait (Brengman and Willems, 2009, p. 351) and a determination as to whether a belief that the low prices for merchandise sold by a store are genuine influences on the perceptions of *Sincerity*.

#### Self-congruity and the shopping conspicuousness situation

Based on the results of the current study, *Public Self-congruity* and *RBI* play crucial roles in the relationship between retail brand personality and positive outcome behaviors. Knowing that the influence of consumers' *Public Self-congruity* on their perceived *overall RBI* could be strengthened by the high *Shopping Conspicuousness Situation*, it is suggested that retail practitioners design environments and execute strategies that attempt to create shopping situations to be perceived as being high in shopping conspicuousness from their customers' perspective. It would thus be more beneficial for retailers to locate their stores in a crowded shopping mall or busy street than in a secluded area. Strategies should also be created in an attempt to encourage customers to patronize a store in the company of shopping companions, such as providing sufficient space for parking, rest areas, restaurants, snack bars, social events, and so on within their stores in order to encourage social interactions between and among shoppers (Yim et al., 2014) or extra discounts and/or provide reward points for bringing co-shoppers to the store (Piskorski, 2011). This is particularly important for discount stores in which the relationship between customers' *Private Self-congruity* and their overall RBI was not significant.

#### The contribution of RBI to behavioral outcomes

The findings of this research also suggest that *RBI* fully mediates the influence of a congruent retail brand personality on behavioral outcomes for all types of retailers. Therefore, retailers, regardless of the formats, should place particular importance on developing memberships and corresponding loyalty programs to foster customers' perceived sense of oneness and belongingness toward specific retail brands. For example, retailers may want to provide more membership activities intended to engage their target customers and deepen their (group) interactions to encourage identifications and in-group favoritism.

### Study limitations

This research had several limitations that should be noted when considering the results and interpreting the findings. For example, the measure of retail brand personality used in this research did not undergo rigorous scale development. The three dimensions might be less representative than the five dimensions identified by the pre-test. The dimension reduction could be a result of fewer traits being included in the main study. To reduce participant fatigue with a long survey, only three traits in each of the five factors with the highest factor loadings were used in the main study. Thus, it is important for future research to develop a synthesized retail brand personality scale that includes more traits and includes other retail formats, such as a specialty store format, on a large consumer sample.

The measures of self-congruity and RBI used in this research were all adapted from the existing literature to fit the retail context investigated in this work. However, the factor structures revealed by the factor analyses in this research were different from those identified in the original sources (Wang and Rao, 1995; Lam et al., 2010; Malär et al., 2011). Future research on related topics in the retail domain should strive to identify scales that have undergone more rigorous scale adaptation procedures. Furthermore, additional measures for RBI that more specifically addresses the development of unique characteristics of retailer shopping environments, such as the tangible experiences gained from interacting with the physical retail environment, the sales personnel, and the other customers in the store setting.

## Data availability statement

The raw data supporting the conclusions of this article will be made available by the author, without undue reservation.

## Ethics statement

The studies involving human participants were reviewed and approved by University of Arizona. The patients/participants provided their written informed consent to participate in this study.

## Author contributions

The author confirms being the sole contributor of this work and has approved it for publication.

## Conflict of interest

The author declares that the research was conducted in the absence of any commercial or financial relationships that could be construed as a potential conflict of interest.

## Publisher's note

All claims expressed in this article are solely those of the authors and do not necessarily represent those of their affiliated organizations, or those of the publisher, the editors and the reviewers. Any product that may be evaluated in this article, or claim that may be made by its manufacturer, is not guaranteed or endorsed by the publisher.
